# Routine Follow-Up Cranial Computed Tomography for Deeply Sedated, Intubated, and Ventilated Multiple Trauma Patients with Suspected Severe Head Injury

**DOI:** 10.1155/2014/361949

**Published:** 2014-01-20

**Authors:** Thomas Erik Wurmb, Stefan Schlereth, Markus Kredel, Ralf M. Muellenbach, Christian Wunder, Jörg Brederlau, Norbert Roewer, Werner Kenn, Ekkehard Kunze

**Affiliations:** ^1^Department of Anaesthesiology, University Hospital of Wuerzburg, Oberduerrbacher Straße 6, 97080 Wuerzburg, Germany; ^2^Department of Intensive Care Medicine, HELIOS Klinikum Berlin-Buch Schwanebecker Chaussee 50, 13125 Berlin, Germany; ^3^Department of Radiology, University Hospital of Wuerzburg, Oberduerrbacher Straße 6, 97080 Wuerzburg, Germany; ^4^Department of Neurosurgery, University Hospital of Wuerzburg, Josef-Schneider-Straße 11, 97080 Würzburg, Germany

## Abstract

*Background*. Missed or delayed detection of progressive neuronal damage after traumatic brain injury (TBI) may have negative impact on the outcome. We investigated whether routine follow-up CT is beneficial in sedated and mechanically ventilated trauma patients. *Methods*. The study design is a retrospective chart review. A routine follow-up cCT was performed 6 hours after the admission scan. We defined 2 groups of patients, group I: patients with equal or recurrent pathologies and group II: patients with new findings or progression of known pathologies. *Results*. A progression of intracranial injury was found in 63 patients (42%) and 18 patients (12%) had new findings in cCT 2 (group II). In group II a change in therapy was found in 44 out of 81 patients (54%). 55 patients with progression or new findings on the second cCT had no clinical signs of neurological deterioration. Of those 24 patients (44%) had therapeutic consequences due to the results of the follow-up cCT. *Conclusion*. We found new diagnosis or progression of intracranial pathology in 54% of the patients. In 54% of patients with new findings and progression of pathology, therapy was changed due to the results of follow-up cCT. In trauma patients who are sedated and ventilated for different reasons a routine follow-up CT is beneficial.

## 1. Introduction

Missed or delayed detection of progressive neuronal damage and secondary brain damage after intracranial injuries may have a negative impact on the outcome of patients with traumatic brain injury (TBI) [1]. Therefore follow-up cranial computed tomography (cCT) has become a diagnostic standard in these patients. [2, 3]. Whether repeated cCT should be done by routine in every patient with TBI or ordered by individual decision remains unclear and is the topic of an ongoing discussion [4–7]. Retrospective data show that routine CT scanning (in the absence of any clinical deterioration) after mild or moderate TBI had no therapeutic (interventional) consequences [6, 8]. On the other hand there is a trend towards routine use for patients with severe TBI but the evidence to support this concept is low. Some authors recommend a cCT scan if clinical signs of neurological deterioration occur [6], other findings suggest that routine cCT might be beneficial in some subgroups of patients [5, 7]. In particular patients with multiple trauma and severe TBI and patients who are endotracheally intubated, mechanically ventilated, and sedated might benefit from routine repeated cCT [5]. To our knowledge there is only a single study which investigated the role of a follow-up cCT scan exclusively in unconscious, sedated, and mechanically ventilated patients with severe TBI [9]. In those patients early clinical signs of neurologic deterioration are potentially difficult to detect. A change in pupils' status, signs of brain herniation, and seizures are commonly clinical signs of severe brain damage and therapeutic intervention might be too late [10].

The timing of the second cCT scan is also not standardized. Recommendations range from 6 to 48 hours after the first scan [10–13]. Over the years the time from accident to the first cCT immediately after admission has decreased continuously. Therefore initial cCT scans might be unremarkable despite intracranial trauma sequel [12, 14]. For that reason some trauma centres schedule the second scan 6 to 24 hours after the admission scan in order to detect early progression of brain injury [12, 15–17].

The objective of this study was to find out whether a routine follow-up cCT 6 hours after the admission scan (as it is the standard of care in our level I trauma centre) is beneficial for deeply sedated and ventilated trauma patients with TBI. We investigated a group of patients with TBI and severe trauma, who had to be sedated and ventilated for the next 48 hours for different therapeutic reasons. Main outcome variables were progression of intracranial injury, new findings in the second cCT scan, and whether these findings resulted in a change of therapeutic management.

## 2. Methods

### 2.1. Setting

The design is a single centre retrospective observational study based on a chart review.

Approval was obtained by the institutional ethic committee.

### 2.2. Diagnostic Procedures in Trauma Patients at the Wuerzburg Level I Trauma Centre

The diagnostic part of our multiple trauma algorithm is based on whole-body multislice computed tomography (MSCT) as the first line diagnostic tool in trauma patients who are supposed to be major trauma victims according to a triage rule [18]. Follow-up cCT is performed routinely within 6 hours after the admission scan in deeply sedated and ventilated trauma patients who are at risk for TBI. Clinical signs for neurologic deterioration trigger an immediate follow-up cCT.

### 2.3. Study Population

For a three-year period trauma patients admitted to the trauma ICU of the Department of Anaesthesiology, University Hospital of Wuerzburg (level I trauma centre), were screened for the following inclusion criteria: trauma patients with TBI who had sedation, intubation, and ventilation for more than 48 hours and who received an initial cCT and a follow-up CT according to the hospital's trauma algorithm.

### 2.4. Exclusion Criteria

Patients with neurosurgical interventions after the first cCT scan were excluded from the analysis.

### 2.5. Data Collection

Two abstractors were trained in the use of the medical charts, the hospital's clinical information system, and the picture archiving and communication system (PACS). Meetings were not scheduled regularly but took place in the case of unexpected problems or questionable data. The investigators carried out spot checks to monitor the performance of the chart abstractors. The reviewers were not blinded.

Patients' data were made anonymous and subsequently entered into a standardized abstraction form. The variables of interest were demographics, Glasgow coma scale, Glasgow outcome scale, the injury severity score (ISS), clinical signs for cerebral deterioration between the first and the second cCT scan, results of the cCT scans, and a change in therapy due to the results of the cCT scan. The results of the cCT scans (defined by a staff radiologist) were checked for the following pathologies: skull fractures, subarachnoid haemorrhage, ventricular bleeding, epidural-subdural and intracranial bleeding (contusion), brain edema, midline shift, and foreign bodies. To compare the findings of the first and the second scan, we defined 4 categories: equal, new findings, progression of known pathologies, and regression of known pathologies. From these 4 categories, we defined 2 groups of patients; group I included those patients with equal or recurrent pathologies and group II included those patients with new findings or progression in pathologies. The change in therapy due to the results of the follow-up cCT is routinely documented in the patients' electronic chart and so could easily be abstracted retrospectively. A change in therapy was classified as follows: evacuation of hematoma, decompressive craniectomy, external ventricular drainage, intracranial pressure monitoring (ICP-monitoring), start of cerebroprotective therapy (deep analgosedation, tight control of oxygen delivery, normoventilation, 30° upright position, and osmotic therapy), and palliative care. Clinical signs for neurological deterioration (e.g., change in pupillary size and reaction, seizures, or a rise in intracranial pressure in patients with ICP-monitoring) were documented in the patients' chart as well.

### 2.6. Statistics

SPSS (SPSS Inc., Il, USA) for windows 15.1 and 17.1 was used to analyse the data. Descriptive data was expressed as means and standard deviations for continuous variables. Fisher's exact test and Mann-Whitney *U*-Test were used to test statistical significance. A value of *P* < 0.05 indicated a statistically significant difference.

## 3. Results

A total of 244 trauma patients were screened for inclusion criteria. 206 patients met the criteria and were included in the analysis. Eight patients died before the second cCT scan. 45 patients had inconclusive or incomplete data and were excluded from the analysis. Three patients had a neurosurgical intervention immediately after the first cCT and were therefore excluded from the analysis. So a total of 150 patients were finally included in the data analysis. All patients were had severe trauma and had initial whole-body MSCT as primary diagnostics.

There were 97 male (64.7%) and 53 female (35.3%) patients; the average age was 39 (±22) years and the average ISS was 25 (±11.4). The reason for prolonged sedation and invasive mechanical ventilation therapy was multiple trauma (*n* = 132, 88%), severe isolated TBI (*n* = 16, 10.7%), and respiratory failure due to pulmonary aspiration of gastric content during the initial trauma.

The GCS on scene was documented in 129 (86%) patients. The mean GCS was 8.9 (±4.6). The mean ICU-stay was 14 (±10.8) days and ventilator days were 12 (±11).

The average time interval between cCT 1 and cCT 2 was 8 (±10) hours while median was 5 hours and 22 minutes (IQR 25–75: 4 hours 25 minutes–6 hours 50 minutes).

31 patients (21%) had clinical signs of neurological deterioration (change in pupil status, *n* = 30, and seizures, *n* = 1) between cCT 1 and cCT 2. None of the patients with intracranial pressure monitoring (*n* = 12) had an increasing intracranial pressure which would have triggered a cCT earlier than 6 hours after the first cCT. In two patients (1%) there were regressive findings in cCT 2 (group I) and in 67 patients (45%) we found no change between cCT 1 and cCT 2 (group I), from which 36 patients had finally a normal cCT 1 and 2. From those patients with a normal CT 1 and CT 2 (*n* = 36) 26 patients had GCS < 15, therefore the diagnosis TBI was justified at the time of admission. From those with initial GCS = 15 (*n* = 10), five patients had severe maxillofacial trauma, one patient had severe scalp avulsion, two patients had neurologic decline before intubation, and one patient had serious trauma of the cervical spine. Therefore the diagnosis of TBI had to be established for those patients. In one patient there were no obvious criteria for the diagnosis of TBI.

A progression of intracranial injury was found in 63 patients (42%) (group II) and 18 patients (12%) had new findings in cCT 2 (group II).

ISS, GCS, and the results of the initial cCT (cCT 1) and the follow-up cCT (cCT 2) are shown in Table 1.

A change in therapy was found in 47 out of 150 patients (31%) and in group II a change in therapy was found in 44 out of 81 patients (54%), respectively. In 19 patients intracranial pressure monitoring was installed, in 5 patients neurosurgical intervention (evacuation of hematoma and decompressive craniectomy) was performed, in 12 patients a cerebroprotective therapy was started, and in 11 patients a change to palliative care was initiated due to futility of care.

On the Glasgow outcome scale, 58 patients (39%) had good recovery (GOS 5), 41 patients (27%) had moderate disability (GOS 4), 23 patients (15%) had severe disability (GOS 3), 5 patients (3%) had a vegetative state (GOS 2), and 21 patients (14%) died (GOS 1) during the hospital stay. The GOS for patients of group I and group II are shown in Table 2.

Clinical signs for neurological decline were found in 31 patients. A change in pupils' status was found in 30 patients (97%) and seizures were found in 1 patient (3%), respectively. From those patients who had clinical signs for neurological decline, 1 patient had new findings and 25 patients had progressive findings in cCT 2. The GOS of those patients is shown in Table 3.

In those patients without clinical signs of neurological deterioration (*n* = 119) there were 55 patients (46%) with progression or new findings on the second cCT. From those patients 24 patients (44%) had therapeutic consequences due to the results of the follow-up cCT.

## 4. Discussion

There is an ongoing discussion over which patients with TBI should undergo routine follow-up cCT. In this retrospective chart review, we wanted to assess whether routine follow-up cCT can reveal significant change in intracranial pathology and whether these findings lead to a change in therapy. We found new diagnosis or progression of intracranial pathology in 54% of the patients. In 50% of patients with new findings and progression of pathology, therapy was changed due to the results of follow-up cCT.

To our knowledge, there is only a single study which focuses exclusively on patients with deep sedation, intubation, and mechanical ventilation [9]. The authors analysed the data of 51 patients with severe TBI, in which one hundred seventeen serial follow-up CTs were performed. None of the CTs resulted in urgent neurosurgical intervention and three scans lead to nonurgent neurosurgical intervention. Looking at the nonsurgical therapeutic consequences, the authors found eleven patients who received invasive intracranial pressure monitoring after a routinely scheduled follow-up scan. So in total, there was a change in therapeutic management in 27% of the patients who received a serial second cCT [9]. This result correlates with our findings, as we found nonsurgical consequences in 26% and evacuation of hematoma and decompressive craniectomy in 11%.

Brown et al. performed a prospective study to identify patients with head injuries who would benefit from routine follow-up cCT [7]. They investigated 274 patients with TBI with a subgroup of patients with severe head injury (*n* = 90). There is no information given whether these patients were sedated and/or intubated for mechanical ventilation. From their data the authors concluded that any head injury with neurologic decline should get a repeat head CT, because one-third of those patients had a change in therapy afterwards. They also concluded that patients with severe TBI (defined as GCS ≤ 8) should undergo routine follow-up cCT, as this might lead to neurological intervention without clinical signs of neurological decline [7]. This approach is supported by Wang and coauthors. In a systematic review of the literature the authors found that studies including patients with severe TBI (GCS 3 to 8) demonstrated a higher incidence of injury progression followed by neurosurgical intervention [5]. Our data also suggests a routine follow-up CT in patients with severe trauma and the need for analgosedation and mechanical ventilation, as neurologic decline is difficult to detect in those patients.

Kaups et al. reported the retrospective data of 462 patients with TBI. They all received a second cCT which showed worsening in 85 (18.4%) patients, from which 16 patients had a change in therapeutic management. An important finding was that every patient with a therapeutic consequence had other clinical findings like hypotension, coagulopathy, ICP elevation, or a worsening of the neurological state. The authors concluded that, in the absence of clinical indicators or risk factors, routine serial follow-up cCT is unnecessary as it does not change the therapeutic management. In contrast to the patients in our study only 68% of the patients were sedated and ventilated, therefore the results have to be compared carefully [6].

A very recent meta-analysis did not demonstrate evidence supporting the management to schedule a follow-up cCT within 24 hours for unchanged or improving patients after mild head injury [19]. The same authors did a single centre study to assess the utility of serial cCT in clinically stable patients with mild TBI and a positive initial cCT. 25 out of 445 patients required intervention, according to the results of the second cCT. All of those had neurological deterioration, and thus the follow-up cCT was performed urgently ahead of schedule (24 hours after the first scan). The authors concluded that routine follow-up cCT in patients (with mild TBI) with unchanged or better neurological status is unnecessary [19]. The main difference to our study is the fact that neurological status is difficult to evaluate in deeply sedated and mechanically ventilated patients. Beginning or slight changes are often difficult to discover, and symptoms like seizures or an alteration in pupil status are late signs of severe progression of injury. Hence, therapeutic interventions might be too late to avoid irreversible secondary brain damage. In our study, 31 patients (21%) had clinical signs of neurological decline (change in pupils state *n* = 30, seizures *n* = 1). 84% of those patients had progressive or new findings in cCT 2. Twenty-two patients (69%) had a change in therapy due to the results of cCT 2. Eighteen patients died (GOS1) and 5 had a severe neurological outcome (GOS 2 and 3). This clearly demonstrates the need for follow-up CT in sedated and ventilated patients with clinical signs of deterioration and highlights the need for close clinical monitoring. On the other hand, there were 55 patients without clinical signs of deterioration with progression of injury and with therapeutic consequences in 24 cases (44%). As demonstrated by Thomas et al. patients with a worsening first cCT have longer hospitalization, higher mortality, and higher chance to require a change in therapeutic management [20]. In our dataset those patients would have been missed when follow-up CT would have been triggered by clinical exam alone.

A systematic review which focused on repeat cCT in patients after blunt head trauma was recently published by Stippler et al. [4]. The authors reviewed 19 studies with a total of 1683 patients. 1630 patients had complicated mild TBI (GCS 13–15 and positive initial CT scan). They found progression of CT findings in 19.9% of the patients. In those patients who had clinical signs for progression of injuries (*n* = 56, 3.4%), worse findings were reported in 67% of the cases. Only 2.4% of the patients had a surgical intervention after the second CT. The authors concluded that repeat cCT is indicated in patients with neurologic decline after mild TBI, as it alters the therapy five times more often than routine follow-up cCT [4].

Main reason to choose the time interval of 6 hours for our protocol was the fact that initial CT might be negative despite relevant intracranial trauma sequels [12, 14, 17]. In addition, many patients with multiple trauma need massive transfusion, suffer from trauma-induced coagulopathy, or require emergency surgery for damage control. Within 6 hours most of these problems might be resolved and at that time it is important to know whether there is progression of intracranial injury, new findings, or a negative follow-up CT. In our opinion the further management of these patients depends strongly on the findings of the follow-up CT. In our study the average time interval for the follow-up CT was 8 hours, while the median was 5 hours and 22 minutes. Goal in daily routine is to obtain the follow-up CT after 6 hours. In certain cases this is not possible. For instance emergency surgery is not finished at that time or the patient needs to be stabilized on the ICU. In other cases emergency surgery is finished earlier than 6 hours. In these cases it might be senseful to get the CT scan on the way back to the ICU in order to minimize transport trauma.

Intrahospital transport of ICU patients might be associated with potentially detrimental complications [21, 22]. For that reason potential benefit needs to be weighed against potential risk. Safe transport can be ensured by establishing an organized and well-prepared management with appropriate equipment and well-trained medical staff [23, 24]. In our department, intrahospital transport of critically ill patients is performed only by ICU staff (physician and nurse). It is a standardized procedure and is performed under full cardiovascular monitoring. In patients with TBI there is a strict protocol that ensures deep sedation, sufficient ventilation, and flat positioning only for the scanning time. Therefore transport of our patients in order to get follow-up CT seems to be justified with a reasonable risk benefit ratio.

In our study we found a low rate of ICP monitoring during the first hours of trauma.

This is well explained by the specific conditions of patients with multiple trauma. For instance, immediate emergency surgery, hemorrhagic shock, or trauma-induced coagulopathy are the reasons for not installing ICP monitoring during the early course of trauma management.

A limitation of our study is that it was carried out based on chart review, since such techniques are prone to bias because of missing, conflicting, or ambiguous data and the subjective nature of some data elements. In order to mitigate some of those disadvantages, strict methodological criteria were used for data collection as described in the method section. Further limitation of the retrospective design of the study is the difficulty to identify what type of therapeutic consequence was really based upon the follow-up CT. Although most changes in therapy were clearly documented in the electronic patient chart, it is difficult to exclude that some changes are based on more data than the results of the follow-up CT alone.

Furthermore the selection of patients with sedation and ventilation for more than 48 hours might be a certain confounder, but on the other hand we wanted to focus exclusively on those patients with severe trauma who require sedation and ventilation for a longer period of time.

As a definition of injury progression, we used the original diagnosis of the radiologist, as this is the relevant basis for therapeutic decisions in the daily routine. Thus we had a clearly defined approach for data interpretation, while interstudy comparisons might be difficult.

## 5. Conclusion

In summary, our data show that routine follow-up CT is beneficial in patients with severe TBI who are sedated and ventilated for different reasons. In patients with a declining neurological status, clinical signs of deterioration are indicators of severe progression of neurological damage and therapeutic intervention might be too late. We therefore recommend a diagnostic approach with the combination of a thorough clinical neurological exam, follow-up CT scan 6 hours after admission, and neuromonitoring as it is considered a standard of care in patients with severe TBI [10].

## Figures and Tables

**Table 1:** Pathologic findings in groups I and II. New and progressive findings in group II.

	Group I (cCT 1) (*n* = 69)	Group II (cCT 1) (*n* = 81)	Group II (cCT 2) progressive findings (% of cCT 1 specific findings)	Group II (cCT 2) new findings (% of group II)
Contusion^†^	*n* = 18 (26%)	*n* = 47 (58%)**	*n* = 14 (30%)	*n* = 15 (19%)
SAH^†^	*n* = 15 (22%)	*n* = 40 (50%)**	*n* = 26 (65%)	*n* = 8 (10%)
SDH	*n* = 8 (12%)	*n* = 10 (12%)	*n* = 5 (50%)	*n* = 3 (60%)
EDH	*n* = 6 (9%)	*n* = 8 (10%)	*n* = 3 (37%)	*n* = 2 (66%)
Ventricular bleeding	*n* = 2 (3%)	*n* = 1 (1%)	*n* = 0	*n* = 1 (1%)
Intracerebral bleeding	*n* = 0	*n* = 5 (6%)	*n* = 4 (80%)	*n* = 2 (2%)
Skull fracture	*n* = 12 (17%)	*n* = 26 (32%)	*n* = 0	*n* = 0
Brain edema^†^	*n* = 4 (6%)	*n* = 17 (21%)*	*n* = 16 (94%)	*n* = 2 (2%)
Midline shift	*n* = 1 (1%)	*n* = 9 (11%)	*n* = 9 (100%)	*n* = 6 (7%)
Foreign body	*n* = 1 (1%)	*n* = 1 (1%)	*n* = 0	*n* = 0

ISS	22 ± 10	28 ± 12*		

GCS	10 ± 4,5	8 ± 4*		

*P < 0.05; *P < 0.001; ^†^Risk for significant worsening in cCT 2.

SAH: subarachnoid haemorrhage; SDH: subdural hematoma; EDH: epidural hematoma; ISS: injury severity score; GCS: Glasgow coma scale; cCT: cranial computed tomography.

**Table 2:** Outcome groups I and II; Glasgow outcome scale (GOS).

cCT results	GOS 1	GOS 2	GOS 3	GOS 4	GOS 5	*∑*
Group I no change	0	0	7	21	39	67
Group I regression	0	0	1	0	1	2
Group II worse	21	5	15	14	8	63
Group II new findings	0	1	2	6	9	18

cCT: cranial computed tomography.

**Table 3:** Clinical signs of neurologic decline and GOS.

Results of cCT 2	Clinical decline	GOS 1	GOS 2	GOS 3	GOS 4	GOS 5
No change	5			2		3
Better	0					
New findings	1				1	
Progressive findings	25	18	2	4	1	

GOS: Glasgow outcome scale; cCT: cranial computed tomography.
